# XGBoost-Based Modeling of Electrocaloric Property: A Bayesian Optimization in BCZT Electroceramics

**DOI:** 10.3390/ma18122682

**Published:** 2025-06-06

**Authors:** Mustafa Cagri Bayir, Ebru Mensur

**Affiliations:** 1 Department of Materials Science and Engineering, Gebze Technical University, 41400 Kocaeli, Türkiye; ebrualkoy@gtu.edu.tr; 2 Volocopter GmbH, 76646 Bruchsal, Germany

**Keywords:** electrocaloric, optimization, electroceramic, regression, XGBoost, processing

## Abstract

Electrocaloric materials, which exhibit adiabatic temperature change under an applied electric field, are promising for solid-state cooling technologies. In this study, the electrocaloric response of lead-free Ba_x_Ca_1−x_Zr_y_Ti_1−y_O_3_ (BCZT) ceramics was modeled to investigate the effects of composition, processing, and measurement conditions on performance. A high-accuracy XGBoost regression model (R^2^ = 0.99, MAE = 0.02 °C) was developed using a dataset of 2188 literature-derived data points to predict and design the electrocaloric response of BCZT ceramics. The feature space incorporated compositional ratios, processing parameters, measurement settings, and atomic-level Magpie descriptors, along with Curie temperature to account for phase-transition behavior. Feature importance analysis revealed that electric field, measurement temperature, and proximity to the Curie point are the most critical factors influencing ΔT_EC_. Bayesian optimization was applied to navigate the design space and identify performance maxima under unconstrained and realistic constraints, offering valuable insights into the nonlinear interactions governing electrocaloric performance. Under room temperature and moderate-field conditions (24 °C, 40 kV/cm), the optimized ΔT_EC_ achieved a value of 1.03 °C for Ba_0.85_Ca_0.15_Zr_0.40_Ti_0.60_, to be processed at 1090 °C for 3 h during calcination, 1300 °C for 2 h during sintering. By integrating experimental insight with machine learning and optimization, this study offers a refined, interpretable framework for accelerating the design of high-performance electrocaloric ceramics while reducing the experimental workload.

## 1. Introduction

The electrocaloric effect (ECE) is a reversible phenomenon in which an applied electric field causes a change in the polarization of a dielectric material, leading to adiabatic variations in temperature and entropy [1,2,3,4]. Electrocaloric materials are being investigated as promising candidates for solid-state cooling, particularly in microelectronic systems where conventional methods like compressor-based refrigeration or convective air and liquid cooling face limitations in size, energy efficiency, and environmental impact. Unlike traditional cooling approaches, electrocaloric systems offer compact integration, silent operation, and avoid the use of harmful refrigerants, making them an attractive alternative for next-generation cooling technologies [4,5,6,7,8].

The efficiency of electrocaloric cooling is influenced by several intrinsic and extrinsic factors, including the material’s ferroelectric properties, phase transition behavior, the strength of the applied electric field, and the measurement temperature. The temperature change (ΔT_EC_) usually reaches its peak near the Curie temperature, where the material transitions from a ferroelectric to a paraelectric phase, accompanied by a significant entropy change due to polarization reordering. As a result, materials with tunable phase transitions near room temperature are especially valuable for practical cooling applications [5,8,9].

Due to environmental regulations and health concerns related to lead-containing materials, lead-free systems have received growing attention. Among them, barium calcium zirconate titanate (Ba_x_Ca_1−x_Zr_y_Ti_1−y_O_3_, or BCZT) has emerged as a promising, environmentally friendly electroceramic with notable electrocaloric performance [10,11,12]. As a solid solution of BaTiO_3_ doped with Ca^2+^ and Zr^4+^, BCZT offers a wide compositional range that enables control over phase transitions, dielectric properties, and polarization behavior. The coexistence and interaction of tetragonal, orthorhombic, and rhombohedral phases in this system support the formation of morphotropic phase boundaries (MPBs), which are known to enhance dielectric, piezoelectric, and electrocaloric responses through domain reconfiguration [10,13,14,15]. Several studies have reported electrocaloric temperature changes exceeding 1 K near MPB compositions of BCZT under moderate electric fields [16,17,18].

Given the advantages of BCZT ceramics, enhancing their electrocaloric performance remains a key research focus. ΔT_EC_ response is highly sensitive to compositional ratios and processing parameters, including calcination and sintering conditions, grain morphology, and domain structure. In addition, measurement conditions, such as the applied electric field, temperature range, and the measurement technique (direct or indirect method, measurement while cooling or heating), introduce further complexity. These interconnected variables create a nonlinear and high-dimensional parameter space, making it difficult to identify and optimize the most influential factors through conventional trial-and-error experimentation.

Data-driven approaches offer an effective way to accelerate the development of high-performance materials [19,20]. By using literature-based datasets and regression models, machine learning can reveal hidden patterns, nonlinear relationships, and complex variable interactions that govern the ΔT_EC_ response. In recent years, such techniques have been widely applied in materials science for tasks like stability prediction, property optimization, and identifying materials descriptors [21,22,23,24,25,26,27,28]. Among these methods, gradient boosting algorithms have shown exceptional performance in regression problems due to their ability to capture intricate feature dependencies through regularization and iterative refinement.

In this study, an XGBoost regression model was utilized to predict the electrocaloric temperature change in BCZT ceramics, employing a literature-based dataset that encompassed detailed information on composition, processing parameters, and measurement conditions. To integrate atomic-scale and phase-transition characteristics, selected Magpie descriptors, such as atomic weight, electronegativity, and DFT-calculated volume, along with Curie temperatures, were incorporated into the feature set. Features with constant values or those unrelated to processing or measurement were excluded to preserve their physical relevance and interpretability. The model demonstrated high predictive accuracy (R^2^ = 0.99), and Bayesian optimization was applied to ascertain the optimal conditions for maximizing ΔT_EC_. This combined approach enables targeted optimization guided by prior experimental knowledge, substantially reducing the experimental workload in electrocaloric material design and processing.

## 2. Materials and Methods

### 2.1. Dataset Generation

**Figure 1:**
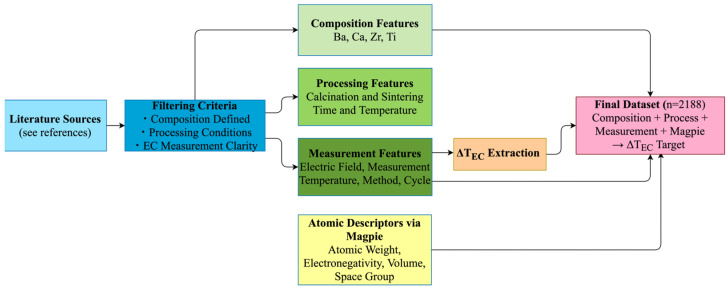
The workflow for dataset generation and feature extraction used in modeling.

To enable accurate predictions of ΔT_EC_ in BCZT ceramics, a comprehensive dataset of 2188 data points was compiled from published experimental studies [1,3,6,10,11,12,13,14,16,17,18,29,30,31,32,33,34,35,36,37]. The dataset focuses on compositions of Ba_x_Ca_1−x_Zr_y_Ti_1−y_O_3_, where electrocaloric measurements are systematically reported. Only entries with clearly defined processing conditions were included, ensuring consistency and reliability across different sources. The workflow to create the dataset is given in Figure 1.

Each data point contains detailed information about the composition of BCZT, thermal processing parameters such as calcination and sintering temperatures, and durations. Curie temperature values were directly extracted from the same experimental studies, and data entries lacking a reported T_C_ value were excluded from the dataset to ensure consistency and physical reliability. To incorporate the thermodynamic proximity to phase transition into the feature space, the difference between the measurement and the Curie temperature (T_C_ − T) was computed. Measurement-related features include the electric field strength, measurement temperature, method (direct or indirect), and whether the measurement was taken during heating or cooling of the sample. To represent these experimental design factors in the model, binary variables were used to encode the measurement mode and polarization stage. ΔT_EC_ values were extracted directly from experimental figures. To enrich the input space beyond stoichiometric ratios, we added atomic-level descriptors using the Magpie feature set, implemented via the matminer library (v0.9.3). Four physically meaningful descriptors were selected: average atomic weight, electronegativity, atomic volume, and space group number. These properties are known to influence dielectric and ferroelectric behavior and possibly help the model detect trends that may not be obvious from experimental data alone. Atomic weight provides insight into lattice mass and vibrational inertia, which in turn affect domain wall mobility. Electronegativity, reflecting the average electron-attracting tendency of constituent atoms, relates to bond polarity and charge localization, both of which are closely linked to polarization stability. The volume per atom serves as an indicator of lattice openness and ionic displacability, influencing how easily entropy can be modulated under an external field. Lastly, the space group number incorporates crystallographic symmetry information, offering indirect cues about structural distortions and phase tendencies that often dictate the strength of the electrocaloric response. Together, these descriptors extend the compositional representation beyond stoichiometry, bringing physically meaningful patterns into the model input space.

### 2.2. XGBoost Regression

The eXtreme Gradient Boosting (XGBoost) regression algorithm was used, which is a technique based on an ensemble of decision trees, where each new tree is trained to correct the prediction errors made by the previous ones. This iterative learning approach allows the model to capture complex and nonlinear interactions between variables, making it particularly suitable for problems involving coupled structure–property relationships such as electrocaloric behavior.

The model was implemented using the scikit-learn (v1.6.1) interface. The dataset was randomly shuffled and then split into 70% for training and 30% for testing, ensuring an unbiased evaluation. In addition to random splitting, a group-aware cross-validation approach was also implemented. In this method, all data entries originating from the same publication were assigned to the same fold, thereby preventing samples from the same research from appearing in both training and test sets. The implications of this approach are further captured in Section 3.

To optimize the model’s performance, Bayesian optimization was performed using the Optuna package (v4.3.0). The objective was to minimize the mean absolute error (MAE) of the predictions. During this process, over 750 different combinations of model settings, known as hyperparameters, were explored. Each combination was evaluated using 5-fold ShuffleSplit cross-validation, which involves repeatedly splitting the training data into different subsets to test the model’s robustness and reduce the risk of overfitting. The optimization was guided by the Tree-structure Parzen Estimator (TPE) algorithm, with Expected Improvement (EI) used as the acquisition function.

The hyperparameters tuned included those controlling the structure of the decision trees, the learning rate of the model, the strength of L1 and L2 regularization terms to prevent overfitting, and the proportions of data and features used in each iteration. Additionally, the number of boosting rounds was optimized between 50 and 1000, and the tree construction strategy was selected from among auto, exact, and approx. and hist, allowing flexibility depending on dataset size and structure. The depth of each tree was carefully constrained to a range of 3 to 5, to keep the model complexity under control. The learning rate was searched within 0.0001 to 1.0 on a log scale, and regularization strengths varied from 10^−8^ to 1.0. At the same time, random sampling and regularization were adjusted to ensure that the model would generalize well to new, unseen data.

After completing the hyperparameter search, the configuration yielding the lowest average MAE across the validation folds was selected. The final model was trained using the entire training set and evaluated on the test set. Standard regression metrics, including mean squared error, mean absolute error, and the coefficient of determination (R^2^), were used to assess performance.

### 2.3. Bayesian Optimizations

To identify the most effective combinations of composition, processing, and measurement parameters that maximize the ΔT_EC_, Bayesian Optimization was applied using the Optuna framework. This approach systematically explores large and complex parameter spaces by building a probabilistic model of the objective function, offering a more efficient alternative to traditional grid or random search methods.

While the XGBoost prediction model included Curie temperature offsets (difference between the measurement temperature and Curie temperature) and Magpie-based atomic descriptors, the optimization phase was deliberately restricted to variables that can be directly controlled in an experimental setting. Therefore, features such as T_C_, T_C_ − T, and Magpie descriptors were excluded from the optimization to ensure that the results reflect realistic and actionable conditions.

The parameter space included the compositional ratios of Ba, Ca, Zr, and Ti; thermal processing conditions; and measurement settings. Stoichiometric constraints were imposed by requiring that the sums of Ba and Ca, and of Zr and Ti, each equal one. Each optimization trial involved a complete set of parameters defining a single sample configuration, and all parameters were bounded by experimentally observed values to maintain physical feasibility.

The tree-structured Parzen Estimator (TPE) algorithm was used to guide the search. This algorithm balances the exploration of lesser-known regions of the parameter space with the exploitation of areas already showing high performance. The optimization aimed to maximize the ΔT_EC_ values predicted by the pre-trained XGBoost model. To avoid unrealistic results, predictions that were negative or physically implausible were penalized heavily during the optimization process. Optimization trials were conducted in 3 stages with 1000, 2000, and 5000 trials to monitor the exploration of the parameter space.

## 3. Results and Discussion

### 3.1. Hyperparameter Optimization and Learning Behavior

The hyperparameter set obtained through Bayesian optimization resulted in a balanced XGBoost model that effectively captures the complex, nonlinear interactions driving the electrocaloric behavior of BCZT ceramics. A total of 750 optimization iterations were performed using the Optuna framework, aiming to minimize the MAE across randomized cross-validation folds.

The best-performing hyperparameter configuration given in Table 1 included a maximum tree depth of 5, which was predefined to mitigate overfitting, which is a common challenge in tree-based models where deeper trees tend to capture noise rather than meaningful structure. The model reached this upper limit, suggesting that moderate tree complexity was sufficient to uncover the structure–property relationships in the dataset. This indicates that although the dataset exhibits rich physical behavior, these patterns can be captured using relatively shallow decision paths.

The learning rate was optimized to a relatively high value of 0.336, enabling rapid convergence and highlighting the existence of strong correlations between the input features and the electrocaloric response. This is further supported by the use of a moderate number of estimators, which balances the model’s ability to learn flexibly while reducing the risk of overfitting commonly associated with high learning rates. Other hyperparameters were jointly tuned to regulate model complexity and improve generalization. The subsample ratio and feature sampling ratio show that nearly the entire dataset and feature space were utilized in each boosting iteration, but with slight randomness to prevent overfitting and enhance robustness. Regularization was applied via both L1 and L2 penalties, providing mild yet effective constraints on tree growth. The gamma parameter, which controls the minimum loss reduction required for a split, was set close to zero, allowing flexible tree development while discouraging uninformative branches.

To account for the skewed distribution of the dataset (high ΔT_EC_ values are rare but critical), the scale positive weight parameter was set to 4.68. This adjustment helps the model place greater emphasis on accurately predicting high-performance samples, which are of scientific and practical interest despite being underrepresented.

Altogether, the optimized hyperparameters reveal a model that is both responsive to meaningful trends and resilient to noise, capturing the complex yet physically traceable relationships governing electrocaloric behavior. The ability to model these relationships with limited tree complexity and minimal regularization suggests that the key mechanisms follow structured patterns that the model can uncover. These results support the effectiveness of XGBoost not only as a predictive tool but also as a framework for revealing insights into the structure-property relations in BCZT ceramics.

### 3.2. Model Performance Evaluation

The regression model demonstrated excellent predictive accuracy in modeling the electrocaloric response of BCZT ceramics. It achieved an MAE of 0.02 °C and an R^2^ score of 0.99 on the test set, indicating a very high agreement between predicted and experimental ΔT_EC_ values. Additional validation using ShuffleSplit (v1.6.1) cross-validation across five randomized folds yielded R^2^ scores between 0.98 and 0.99, with an average of approximately 0.988, confirming the model’s robustness and consistency across different data subsets. The model performance was also evaluated using a group-aware cross-validation strategy. This approach led to a reduction in predictive accuracy, with MAE increasing to 0.12 °C, as the model is no longer exposed to closely correlated data from the same source during both training and testing. However, the key feature trends remain stable across both validation schemes, and this consistency supports the model’s insights. A detailed for the comparison of grouped versus shuffled validation is outlined in the future work section.

**Figure 2:**
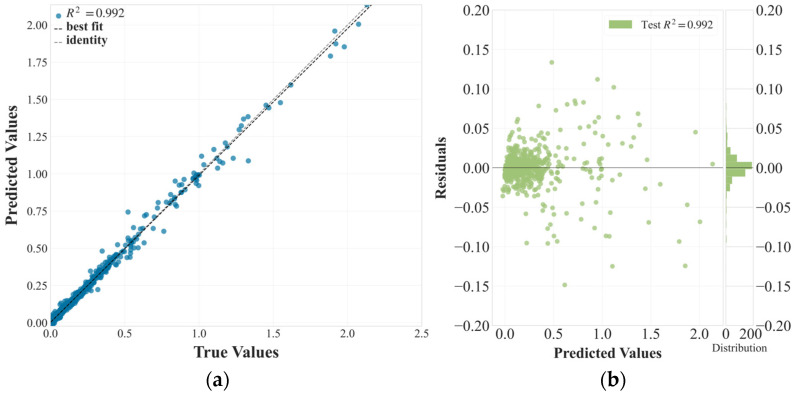
Evaluation of XGBoost model performance for ΔT_EC_ prediction. (**a**) Predicted versus true ΔT_EC_ values, (**b**) prediction residuals (difference between predicted and true values).

Figure 2 presents the model’s performance using two complementary visualizations. The prediction error plot (Figure 2a) shows predicted versus actual ΔT_EC_ values, with most points clustered tightly along the identity line. This indicates that the model successfully captures the underlying relationships between composition, processing, and measurement parameters and the resulting electrocaloric effect.

The residuals plot (Figure 2b) provides further insight into model behavior. Residuals are symmetrically distributed around zero and show no systematic trends, suggesting that the model does not suffer from structural bias. Most residuals fall within a narrow ± 0.05 range, supporting the model’s ability to make reliable predictions across the full range of ΔT_EC_ values. A small number of outliers appear at higher ΔT_EC_ values as an expected outcome in physical regression tasks where nonlinearity or sharp thresholds may influence material responses. These deviations may reflect extrapolation limits in rarely sampled compositions or processing conditions.

The strong performance of the model can be attributed to several key factors: the inclusion of experimentally relevant processing parameters as well as the entire trends of the electrocaloric behavior throughout the measurements, the addition of atomic-scale Magpie descriptors, the use of Curie temperature as a physical indicator, and the thorough optimization of hyperparameters through Bayesian tuning.

To benchmark the chosen XGBoost model against other machine learning approaches, additional models, including Random Forest, Support Vector Regression (SVR), Multi-Layer Perceptron (MLP), decision tree, and Lasso regression, were trained and evaluated on the same dataset. Random Forest and SVR achieved comparably low MAE values (~0.05 °C). These models, particularly Random Forest, are well-suited to capturing local patterns and decision boundaries. However, they might lack the global regularization and feature interaction modeling that gradient-boosted models like XGBoost provide.

MLP and Lasso yielded higher prediction errors of 0.10 °C and 0.11 °C, respectively. The performance of MLP can be attributed to its sensitivity to data volume and hyperparameter complexity. Lasso regression, which assumes linearity and applies L1 regularization for automatic feature selection, offers model simplicity but fails to capture the nonlinear dependencies and coupled interactions intrinsic to electrocaloric response. Altogether, XGBoost demonstrates a more favorable balance between predictive accuracy, robustness, and interpretability.

Compared to previous machine learning studies on electrocalorics, the present work contributes an expanded dataset and a complementary modeling approach. Gong et al. employed XGBoost on a smaller dataset and reported a test MAE of 0.14 °C with an R^2^ score of 0.91 [24]. Su et al. utilized a neural network architecture on approximately 1800 data points and obtained an MAE of 0.07 °C, although without incorporating features such as Curie temperature or atomic-level descriptors [23]. The model performance of the present work highlights the potential benefits of integrating domain-informed features with optimized gradient boosting techniques. While differences in dataset construction and evaluations should be considered, these results collectively reinforce the utility of machine learning approaches for electrocaloric property predictions.

### 3.3. Feature Importance and SHAP Analysis

**Figure 3:**
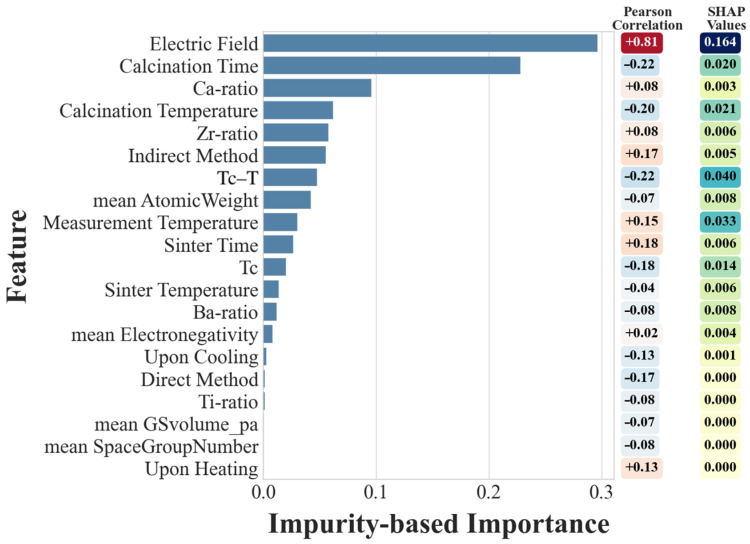
Feature importance analysis for ΔT_EC_ prediction using impurity-based ranking, SHAP values, and Pearson correlation.

To identify the key factors influencing the electrocaloric response of BCZT ceramics and to understand how the XGBoost model makes predictions, a combined feature importance analysis was conducted using impurity-based rankings and SHAP (SHapley Additive exPlanations) values. The impurity-based method measures how frequently and effectively a feature is used in decision tree splits to reduce prediction error. While this approach can favor continuous variables with broad ranges, it remains a powerful tool for constructing initial feature hierarchies in tree-based models. According to the impurity-based importance results as shown in Figure 3, the electric field is by far the most dominant factor governing ΔT_EC_.

This aligns with electrocaloric theory, where the external field directly drives dipole reorientation, entropy change, and temperature shift in ferroelectric materials [8]. The second and third most influential variables are calcination time and Ca ratio, which highlight the critical role of processing conditions and compositional balance in determining the material’s microstructural evolution and polarization behavior. Specifically, Ca substitution in the A-site of the perovskite structure lowers crystallographic symmetry and promotes relaxor behavior, enhancing local polarization fluctuations and thus increasing the entropy change under an external field [38]. Other important contributors include calcination temperature, Zr ratio, and the indirect method label, suggesting that both thermal treatment and measurement mode significantly affect the electrocaloric performance. While T_C_ − T (the difference between Curie temperature and measurement temperature) still holds relevance, its impact appears more moderate than initially expected, based on the impurity-based analysis.

These findings emphasize that ΔT_EC_ is not governed by a single parameter but rather emerges from a coupled influence of applied field, thermal history, and compositional tuning. Processing parameters such as calcination time and temperature may influence grain growth and domain wall mobility, and elemental Ca and Zr ratios control phase stability [12,15]. All these factors collectively influence the ferroelectric behavior of the material through their effects on domain configuration, phase composition, and polarization stability. Among these, a higher Ti content increases tetragonal distortion and enhances spontaneous polarization in the BCZT lattice, thereby amplifying the entropy change under electric field application. Conversely, Zr incorporation can stabilize rhombohedral or orthorhombic phases, which affects the phase transition characteristics and domain dynamics, contributing to the observed electrocaloric response [30,39].

**Figure 4:**
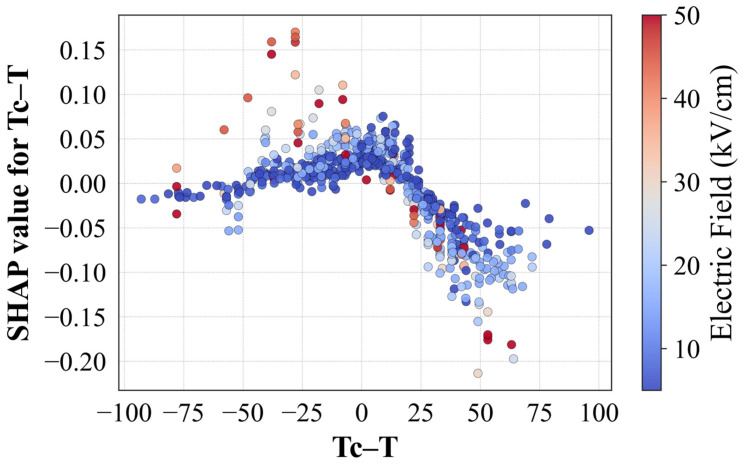
SHAP dependence plot for T_C_ − T, colored by electric field.

SHAP analysis was employed to provide a more interpretable, instance-level understanding of how features contribute to individual predictions to complement global rankings. SHAP values can be seen on the right side of Figure 3. These values are particularly useful in revealing feature interactions and nonlinear effects, such as the diminishing influence of the electric field at saturation or the bell-shaped behavior of ΔT_EC_ near the Curie temperature. The electric field showed a generally linear positive influence on ΔT_EC_ up to moderate field levels, after which a saturation trend was observed—an expected physical phenomenon linked to the limits of dipole reorientation. For T_C_ − T, SHAP analysis revealed a parabolic relationship given in Figure 4, with maximum ΔT_EC_ contributions occurring just below the Curie temperature. This behavior is explained by the maximized dielectric susceptibility and entropy reconfiguration near the phase transition point, where ferroelectric domains become highly reconfigurable under applied fields. At temperatures far below or above T_C_, the response diminished due to limited domain mobility or the absence of spontaneous polarization, respectively. This trend underlines the necessity of tuning measurement temperature near the phase transition point to maximize performance. Both impurity-based and SHAP analyses identify that optimal behavior emerges from a careful balance of compositional tuning, thermal processing, and measurement setup.

### 3.4. Bayesian Optimization for Electrocaloric Performance Enhancement

A Bayesian Optimization framework was implemented to identify optimal combinations of compositional, processing, and measurement parameters that maximize the ΔT_EC_. This method efficiently explored a high-dimensional parameter space by iteratively improving the search based on previous evaluations, making it suitable for complex material design problems. The optimization was conducted in stages, and the achieved processing parameter sets are summarized in Table 2, including the predicted ΔT_EC_ values (in °C), electric field levels (in kV/cm), temperatures (in °C), and process durations (in hours).

In the first phase, no constraints were imposed on the electric field or measurement temperature, allowing full exploration across their experimental ranges. Notably, after 2000 trials, the optimization stabilized around a consistent maximum, and after 5000 iterations, the optimization converged to a maximum predicted ΔT_EC_ of 2.15 °C. This was achieved with a Ba-rich composition of Ba_0.79_Ca_0.21_Zr_0.29_Ti_0.71_, calcined at 1058 °C for 5 h and sintered at 1385 °C for 10 h. The optimal measurement conditions were determined as an electric field of 88 kV/cm and a temperature of 105 °C, using the direct method during heating. These findings indicate a pronounced response region at elevated temperatures and strong electric fields, where phase transition-driven entropy change is maximized. The results highlight the significant electrocaloric potential of BCZT ceramics under high-field and elevated-temperature conditions.

Recognizing that such conditions may exceed practical experimental limits—particularly due to the risk of dielectric breakdown—subsequent optimization stages introduced constraints. Therefore, in the second phase, the measurement temperature was fixed at 24 °C to simulate room-temperature device operation. Within 1000 optimization trials, the best configuration yielded a ΔT_EC_ of ~1.14 °C, using a composition of Ba_0.53_Ca_0.47_Zr_0.32_Ti_0.68_, calcined at 1019 °C and sintered at 1371 °C, both for 3 h. The electric field to be applied is 39.3 kV/cm, which is significantly lower than in the unconstrained scenario but still yields a viable electrocaloric response. When the number of trials was increased to 2000, the maximum ΔT_EC_ improved only marginally (~0.0006 °C), and the optimal composition remained structurally similar. However, by 5000 trials, the optimizer identified a distinct configuration with a higher ΔT_EC_ corresponding to a different Ba_0.86_Ca_0.14_Zr_0.38_Ti_0.62_ composition.

**Figure 5:**
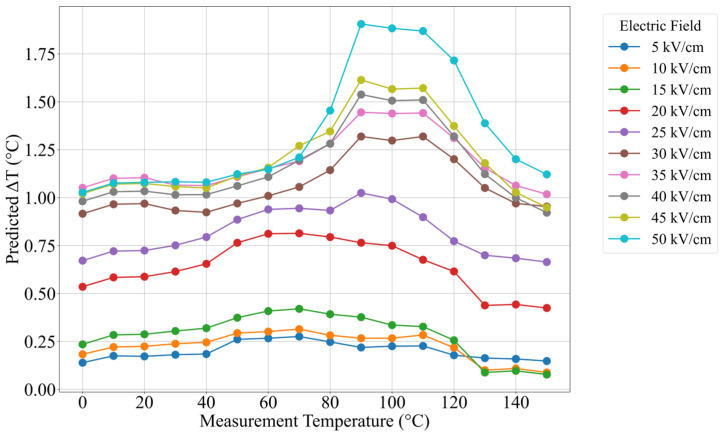
Predicted ΔT_EC_ response curves as a function of measurement temperature for various electric field strengths.

**Figure 6:**
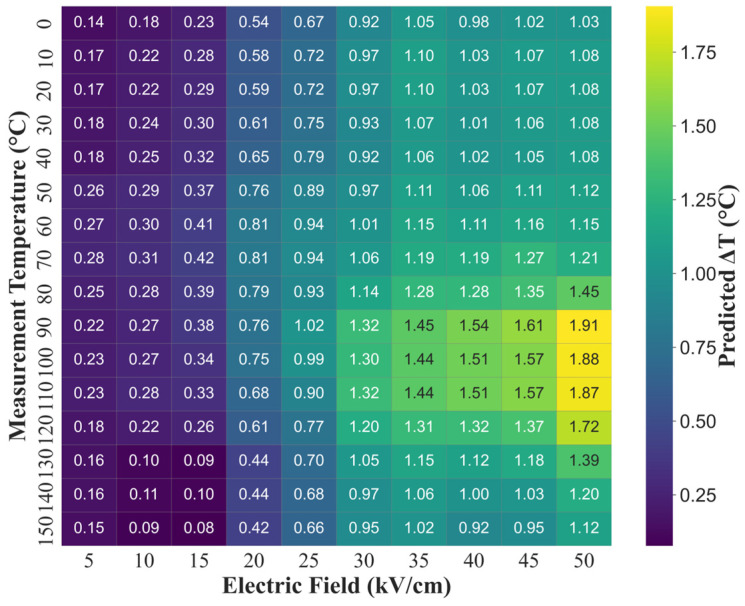
Heatmap of predicted ΔT_EC_ values as a function of measurement temperature and electric field strength.

To visualize the response behavior, ΔT_EC_ values were predicted across a grid of measurement temperatures (0–150 °C) and electric field strengths (5–50 kV/cm). The line plot given in Figure 5 illustrates the ΔT_EC_ response curve for each electric field, revealing a peak in performance around 90–100 °C, close to the Curie temperature. This behavior is consistent with the known thermodynamics of the electrocaloric effect, where entropy change and dipolar reordering are maximized near the transition point [8]. The heatmap in Figure 6 shows a different visualization of the optimized BCZT, highlighting the concentrated performance region within the 90–100 °C and 35–50 kV/cm. The ΔT_EC_ response starts to increase field strength beyond 30 kV/cm. The map also reveals a relatively flat region at low fields (<15 kV/cm), where ΔT_EC_ remains below 0.5 °C regardless of temperature, indicating the need for sufficient polarization driving for the electrocaloric response. These patterns validate the physical reliability of the trained XGBoost model and confirm that it successfully captures the relations and can serve as a performance prediction and optimization for finding the maximized cooling potential.

## 4. Conclusions

This study presents a robust, interpretable machine-learning framework for accelerating the discovery and optimization of electrocaloric (EC) materials. By training a regression model on a literature-derived dataset enriched with atomic descriptors and Curie temperature data, the approach accurately predicted the ΔT_EC_ in BCZT ceramics. SHAP analysis confirms that the electric field strength, temperature proximity to the Curie point (T_C_ − T), and measurement temperature are primary contributors to the EC response, with processing conditions also playing a significant role.

Bayesian Optimization efficiently identifies high-performance material–process–measurement combinations under both ideal and experimentally realistic constraints. While the theoretical maximum ΔT_EC_ of 2.15 °C is achieved under unconstrained conditions, the model also yields a strong performance (>1 °C) under standard laboratory setups, demonstrating its practical relevance.

Beyond prediction, the framework offers actionable experimental guidance by mapping complex, nonlinear interdependencies, and narrowing feasible design spaces. This work combines a large-scale, the literature-derived database with explainable machine learning and physically grounded optimization. It thus offers a comprehensive strategy for materials-by-design approaches in electrocaloric ceramics. Altogether, the findings present a coherent, data-driven framework for intelligent EC material design, leading to future advancements that integrate microstructural features and multi-objective optimization strategies.

Additionally, a group-aware cross-validation was applied to ensure that all data from the same study remained within a single fold, avoiding overlap between training and test subsets. While this reduced predictive accuracy due to the exclusion of intra-study similarities, key feature trends remained stable, reinforcing the model’s robustness. A comparative study exploring validation strategies and generalization behavior is planned as future work.

## Figures and Tables

**Table 1:** Optimum hyperparameters found XGBoost model after grid search.

Hyperparameter	Value
Number of estimators	195
L1 regularization weight	0.0030
L2 regularization weight	5.3 × 10^−8^
Subsample ratio	0.913
Feature sampling ratio	0.963
Maximum tree depth	5
Minimum child weight	1
Learning rate	0.336
Gamma	1.6 × 10^−6^
Scale positive weight	4.68
Tree method	Auto

**Table 2:** Summary of process parameter sets across Bayesian optimization trials. Bold values shows the fixed values in the optimization cycle.

Trials	Ba	Zr	T_calc_	t_calc_	T_sint_	t_sint_	E	T	Method	Cycle	ΔT_EC_
1000	0.73	0.28	1077	5	1384	10	87	106	Direct	Heating	2.14
2000	0.78	0.29	1058	5	1385	10	88	105	Direct	Heating	2.15
5000	0.78	0.29	1058	5	1385	10	88	105	Direct	Heating	2.15
1000	0.51	0.34	1019	3	1371	3	39	** 24 **	Direct	Heating	1.14
2000	0.51	0.34	1062	3	1359	2	38	** 24 **	Direct	Heating	1.14
5000	0.86	0.37	1024	3	1352	2	38	** 24 **	Direct	Heating	1.14
1000	0.85	0.39	1090	3	1300	2	** 40 **	** 24 **	Direct	Heating	1.03
2000	0.85	0.39	1090	3	1300	2	** 40 **	** 24 **	Direct	Heating	1.03
5000	0.85	0.39	1090	3	1300	2	** 40 **	** 24 **	Direct	Heating	1.03

## Data Availability

The raw data supporting the conclusions of this article will be made available by the authors on request.
